# Applications of artificial intelligence in orthodontics: a bibliometric and visual analysis

**DOI:** 10.1007/s00784-025-06158-y

**Published:** 2025-01-16

**Authors:** Alessandro Polizzi, Mattia Boato, Sara Serra, Vincenzo D’Antò, Rosalia Leonardi

**Affiliations:** 1 Department of General Surgery and Surgical-Medical Specialties, Section of Orthodontics, University of Catania, Via S. Sofia 68, Catania, 95124 Italy; 2 Department of Neurosciences, Reproductive Sciences and Oral Sciences, Section of Orthodontics, University of Naples “Federico II”, via Pansini, 5, Naples, 80131 Italy

**Keywords:** Artificial intelligence, Deep learning, Cephalometric analysis, Bibliometric analysis, Orthodontics, Machine learning, Segmentation

## Abstract

**Objectives:**

To conduct a comprehensive bibliometric analysis of the literature on artificial intelligence (AI) applications in orthodontics to provide a detailed overview of the current research trends, influential works, and future directions.

**Materials and methods:**

A research strategy in The Web of Science Core Collection has been conducted to identify original articles regarding the use of AI in orthodontics. Articles were screened and selected by two independent reviewers and the following data were imported and processed for analysis: rankings, centrality metrics, publication trends, co-occurrence and clustering of keywords, journals, articles, authors, nations, and organizations. Data were analyzed using CiteSpace 6.3.R2 and VOSviewer.

**Results:**

Almost 83% of the 381 chosen articles were released in the last three and a half years. Studies were published either in highly impacted orthodontic journals and also in journals related to informatics engineering, computer science, and medical imaging. Two-thirds of the available literature originated from China, the USA, and South Korea. AI-driven cephalometric landmarking and automatic segmentation were the main areas of research.

**Conclusions:**

This report offers a thorough overview of the AI current trend in orthodontics and it highlights prominent research areas focused on increasing the speed and efficiency of orthodontic care. Furthermore, it offers insight into potential directions for future research.

**Clinical relevance:**

Collaborative research efforts will be necessary to strengthen the maturity and robustness of AI models and to make AI-based clinical research sufficiently reliable for routine orthodontic clinical practice.

## Introduction

Dentistry and orthodontics have seen significant changes during the past few decades due to a shift from analog to digital that has led to a computerized workflow [1].

In this regard, the advent of artificial intelligence (AI) has ushered in a transformative era across various medical disciplines, including orthodontics [2]. AI technologies, encompassing machine learning (ML), artificial neural networks (ANNs), and deep learning (DL) are poised to revolutionize the way how healthcare is delivered, offering unprecedented advancements in diagnostic speed, accuracy, treatment planning, and overall patient outcomes. As these technologies evolve, their integration also into orthodontic practice promises to enhance clinical efficiency and, what’s more important, to personalize treatment modalities, thereby improving patient satisfaction and success rates [3].

In orthodontics, the potential AI applications in the future may be vast and varied. From automating diagnostic processes to predicting treatment outcomes and customizing appliance design, AI could reshape traditional methodologies [4]. AI in orthodontics has been the subject of an increasing number of research and projects in recent years to evaluate if algorithms may integrate some aspects of clinical practice. Due to the rapid development of AI technology and the wide spectrum of applications, the main research direction is not yet clear [3].

Thus, given the fast development of AI in oral health, it is advisable to systematically assess the current state of orthodontics research to evaluate the application and future trends with a fully comprehensive quantitative analysis such as bibliometrics [5].

Bibliometric analysis is a popular method in medical research since it is simple, reliable, and effective as it involves explorative data analysis and an advanced study of co-occurrences, co-authorship, citation burst, and thematic evolution [6, 7]. In particular, compared to descriptive reviews, bibliometric analysis involves the quantitative evaluation at both macro- and micro-levels in target scientific fields of publication patterns, citations, influential studies, past and future key trends, activities, and cooperations among authors and institutions, and research impact. Bibliometric analysis, using software such as CiteSpace and VOSviewer, may help illustrate inter-relationships between these different kinds of information through knowledge mappings [8–10]. By examining the volume, growth, and influence of publications, as well as identifying key researchers, institutions, and journals contributing to this area, bibliometric analysis can uncover the dynamics shaping the field, highlight emerging trends, and overcome research gaps [11].

Therefore, this kind of approach may provide a comprehensive method to identify future research key directions on AI use in orthodontics. In this regard, a bibliometric study on AI in dentistry [12] showed that the analysis of imaging data has been the main emphasis of AI in dentistry, and the dental conditions most commonly linked to AI were periodontitis, bone fractures, and dental caries. However, the only previous bibliometric analysis [13] focused on the use of AI in orthodontics and craniofacial surgery by including the top-cited articles just published in dental journals. This kind of approach can lead to underestimate more recent papers because even if of importance did not have the time to be quoted. Moreover, in that search [13], not all journals in the fields of informatics and engineering were included. In this regard, this study is intended to bring a comprehensive quantitative analysis of the literature available on the use of AI in orthodontics, without quoting limitations.

Therefore, this study aims to carry out a comprehensive bibliometric analysis of the literature on AI studies in orthodontics to provide a detailed overview of the current research trends, influential works, and future directions.

## Materials and methods

CiteSpace and VOSviewer were the two software we used in the present study.

### Data source

The Web of Science (WoS) Core Collection at the electronic library of the University of Catania was searched with the following query: ((Orthodontic* OR cephalometr* OR cervical vertebrae) OR (segmentation AND (tooth OR teeth OR mandible OR maxilla OR airway*))) AND (deep learning OR artificial intelligence OR machine learning OR artificial neural network* OR convolutional neural network*) covering the publication period between 01-01-1985 to 28-06-2024. The search language was set to English.

### Data screening

Two separate researchers (M.B. and S.S.), screened the records based on titles and abstracts, and the studies that did not meet the inclusion criteria were excluded. Articles were included if: (1) relevant to the use of AI in orthodontics; (2) randomized clinical trials (RCTs), non-randomized clinical trials (nRCTs), and observational studies (cross-sectional, cohort, and case-control), prospective or retrospective. The following exclusion criteria were considered for the selection of eligible articles: (1) off-topic studies not employing AI in orthodontics; (2) narrative and systematic reviews; (3) opinion articles, editorials, conference reports, and case reports/series; (4) books; and (5) retracted publications. After the screening, the two researchers reached a Cohen’s kappa agreement score of 0.91, indicating a high degree of agreement. Disagreements between the reviewers over the final selection of some articles were solved with the involvement of a third author (A.P.) and full-text evaluation if necessary. The following data were extracted from the WoS database: the total number of citations and publications, WoS categories and topics, journals, countries, keywords, authors, affiliations, and funding organizations. Full counting of these data was taken into account.

### Data import and processing

The final set of included literature was imported in plain text format into CiteSpace 6.3.R2 software (Dressel University, PA, United States). CiteSpace was developed for the visualization of emerging trends and progressive knowledge domains [14]. Both software analysis and manual analysis were performed in the data processing. The first literature analysis was conducted on CiteSpace 6.3.R2 to assess rankings, centrality metrics, and co-occurrence and clustering of keywords for authors, nations, and organizations. The final program parameters for bibliometric analysis were as follows: “Time period = 2005–2024,” “Year Slice = 1,” “g-index = 25,” and “Top N% = 10.” All the other parameters were selected to default.

VOSviewer makes it possible to create bibliometric networks that illustrate interactions between various entities such as publications, keywords, institutions, or researchers. Furthermore, co-authorship analysis, bibliographic coupling, and co-citation generation are supported by VOSviewer. While CiteSpace offers unique statistical features, VOSviewer is a better tool for visualizing keyword co-occurrences.

Thus, VOSviewer 1.6.19.0 (Leiden University, Holland) was employed to optimize the visualization of results [15, 16]. Bibliometric analysis was conducted based on the indications reported in the VOSviewer user manual [17]. In particular, VOSviewer may develop bibliometric networks and is used for data documentation, co-occurrence clustering, and visualization. In the present study, the co-occurrence networks of keywords, journals, authors, countries, and collaborations were processed and illustrated with this software. The quantity of co-occurrence frequencies between two things determines the link strengths between the co-authorship, region cooperation, and keyword co-occurrence networks; the overall link strength is the sum of all linkages between an item and other items.

### Data analysis and indicators

The results of this study are mostly shown as numbers and percentage representations together with visual network maps. CiteSpace was used for basic literature analysis, clustering, and burst analysis to identify recurrent keywords. The analyses included impact networks (references and journals), contribution and collaboration networks (authors, nations, and institutions), and keyword analysis (cluster). The top 15 keywords with the strongest citation bursts were analyzed. Moreover, to assess the output’s quality, three parameters were employed: silhouette, modularity, and centrality. To determine the significance of a node, especially one that occupies a central position in a cluster or acts as a bridge, centrality computes the shortest pathways between every pair of nodes in the network [18]. Keyword clustering can highlight linked study areas and their evolution over time by grouping strongly correlated nodes together [19]. Modularity, or Q score, establishes the quality of the cluster division in the network and ranges from 0 to 1: a Q score greater than 0.3 denotes a well-organized network. Lastly, the quality of the clustering arrangement was evaluated with the silhouette score (S score), which ranges between − 1 and + 1. Whether a network’s S score is more than 0.3, 0.5, or 0.7 indicates it is homogeneous, acceptable, or very reliable [20]. The top 10 cited journals and articles, the top 9 authors, the top 12 nations, and the top 6 WoS categories according to the number of published articles were reported in terms of counts. Moreover, graphic representations of publication trends were created.

## Results

**Fig. 1:**
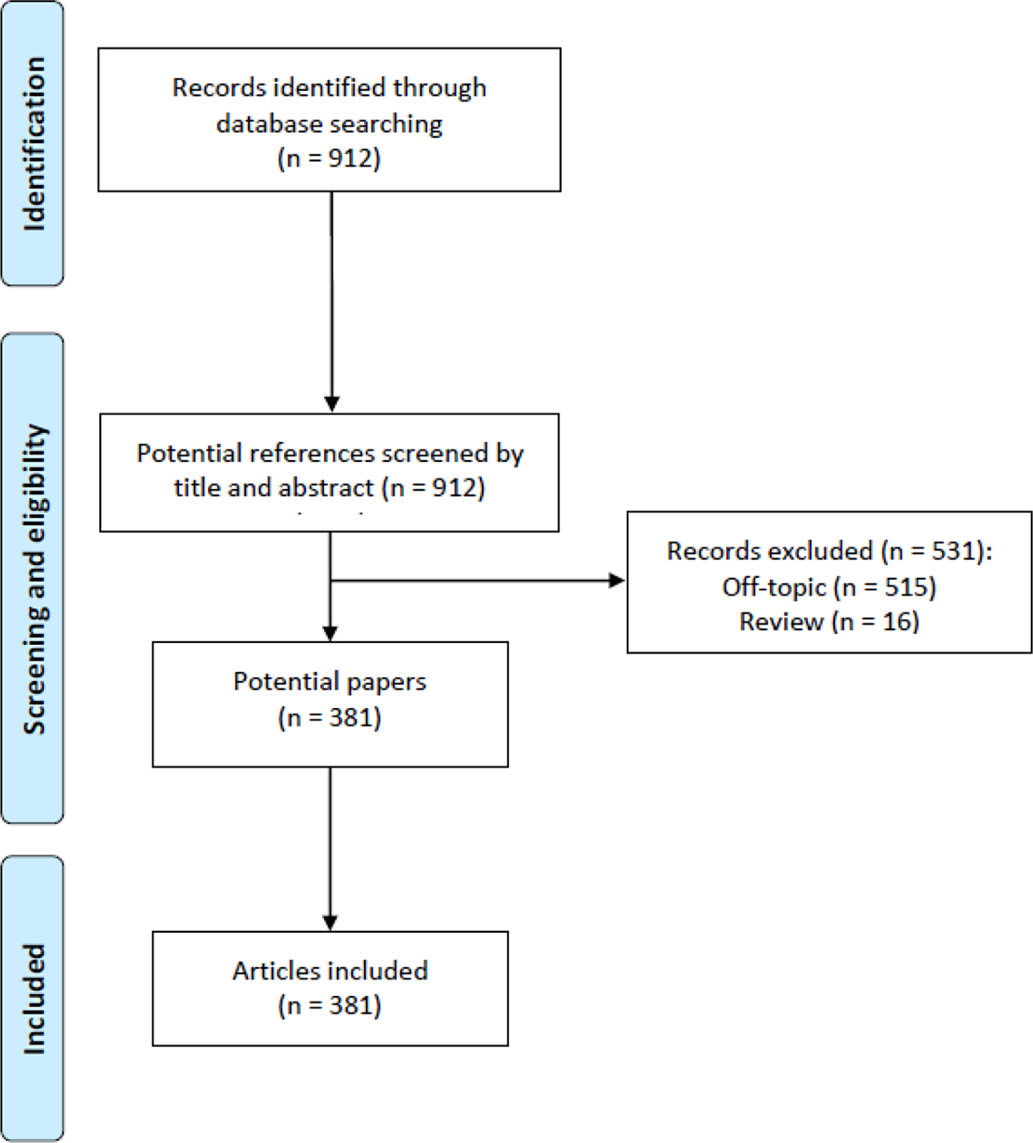
Flow chart of the study

Based on the research protocol, 912 records, including only the “articles” section in WoS research, have been identified and then screened by title and abstract for their adherence to the main topic of this study. After this process, 531 records were excluded because not related to the use of AI in orthodontics (*n* = 515), or for the study design as review (*n* = 16). Finally, 381 articles were included in the present bibliometric analysis (Fig. 1).

### Annual publication trend

**Fig. 2:**
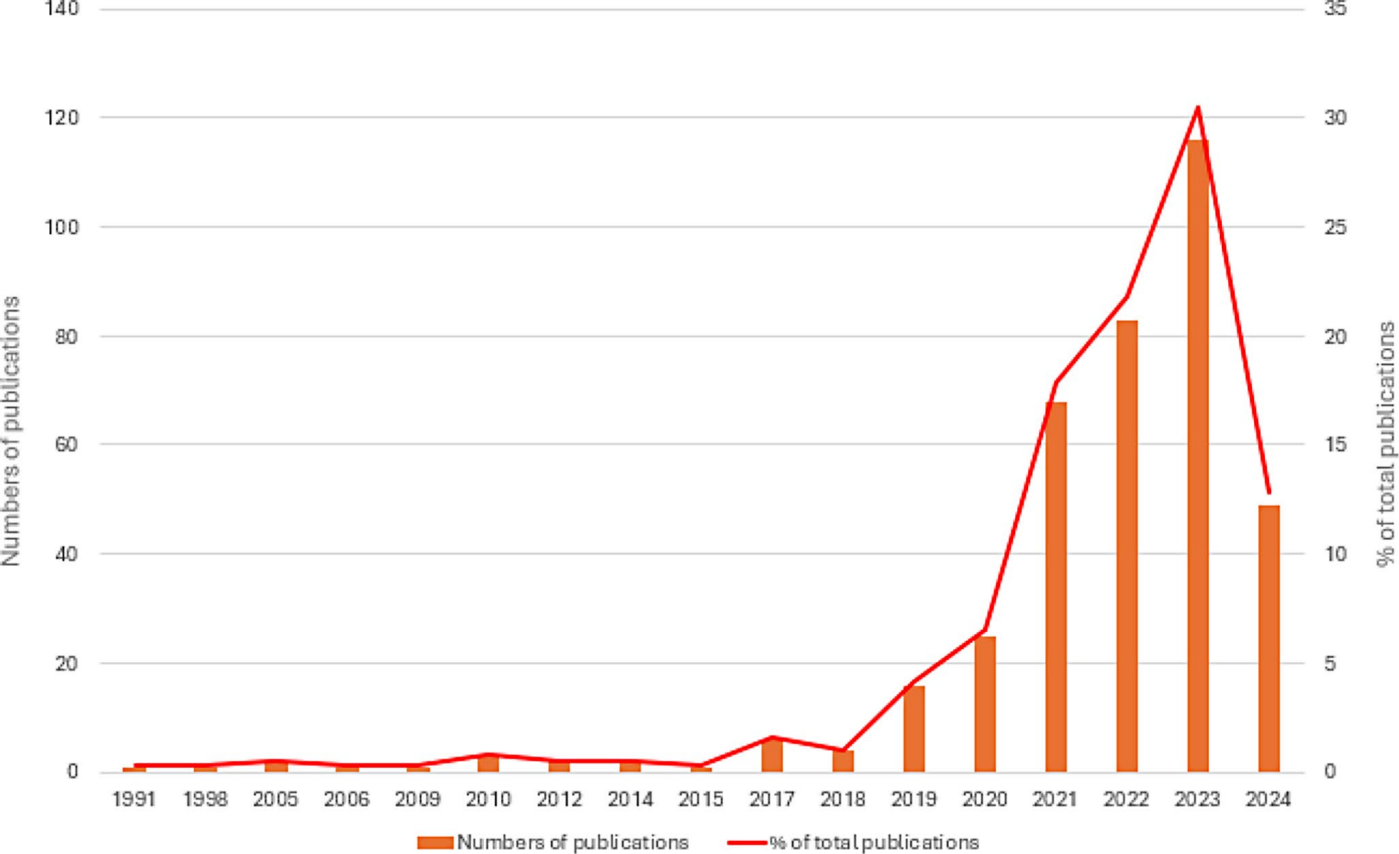
Publication trend from 1991 to 2024. The vertical bars indicate the absolute number of publications, whereas the red line represents the percentage proportion

Figure 2 presents the publication trend of the included articles. The peak of publication was reached in 2023 with 116 articles (30,44% of the total publications). Interestingly, the last 316 articles (82,92% of the total available literature on the topic) were published in the last three and a half years (range 2021–2024). This may be due to the progressive interest of the scientific community and the new frontiers opened in the use of AI models in orthodontics. In the publication trend for 2024, it should be taken into account that the research is updated to 28th June and that only after the end of the year it will be possible to have a definitive picture of the current year’s trend.

### Journal analysis

As shown in Table 1, the top 3 most influential journals for the number of citations were the *American Journal of Orthodontics and Dentofacial Orthopedics*, *The Angle Orthodontist*, and the *Lecturer Notes in Computer Science*. These 3 journals were the leading journals in the field of AI in orthodontics with the following centrality scores respectively: 0.25, 0.13, and 0.10. In particular, *The Angle Orthodontist*, the *American Journal of Orthodontics*,* and Dentofacial Orthopedics* and *Diagnostics* were the three journals with the highest total link strength. The most impactful WoS categories involved in the field of AI in orthodontics have been listed in Table 2. It is interesting to observe that, although Dentistry and Oral Surgery is the first category with a total of 165 counts, Biomedical Engineering, Medical Imaging, Electronic Engineering, and Computer Science also provided a substantial contribution to the topic development as shown by their counts and centralities values.

**Table 1:** Most cited journals for the use of AI in orthodontics

No	Count	Centrality	Year	Cited journals
1	222	0.25	1998	American Journal of Orthodontics and Dentofacial Orthopedics
2	209	0.13	2005	The Angle Orthodontist
3	173	0.10	2005	Lecture Notes in Computer Science
4	160	0.03	2019	Scientific Reports UK
5	154	0.08	2005	European Journal of Orthodontics
6	151	0.08	2005	IEEE Transactions on Medical Imaging
7	149	0.04	2017	Conference on Computer Vision and Pattern Recognition (CVPR) IEEE
8	146	0.04	2017	Medical Image Analysis
9	138	0.04	2014	Dentomaxillofacial Radiology
10	110	0.03	2017	ARXIV

**Table 2:** Top 10 WoS categories involved in the publication of articles on the use of AI in orthodontics

No	Count	Centrality	Year	Cited journals
1	165	0.06	2010	Dentistry, Oral Surgery & Medicine
2	47	0.55	2005	Engineering, Biomedical
3	42	0.05	2014	Radiology, Nuclear Medicine & Medical Imaging
4	36	0.41	2015	Engineering, Electrical & Electronics
5	35	0.00	2021	General & Internal Medicine
6	28	0.13	1991	Computer Science, Interdisciplinary Applications
7	25	0.13	2005	Computer Science, Artificial Intelligence
8	24	0.05	1991	Computer Science, Information Systems
9	24	0.00	2018	Multidisciplinary Sciences
10	15	0.00	2019	Telecommunications

### Reference analysis

The top 10 articles classified for number of citations are presented in Table 3. It is interesting to underline that the first article for the number of citations (190) by Miky et al. [21], focused on the use of AI for automatic teeth classification in cone-beam CT, whereas the second and third classified by Arik et al. (153 citations) [22] and Park et al. (106 citations) [23] respectively regarded the use of convolutional neural networks and deep learning for automatic cephalometric analysis.

**Table 3:** Top 10 references for number of citations

No	Citations	Article	Year	Author
1	190	Classification of teeth in cone-beam CT using deep convolutional neural network	2017	Miky Y et al.
2	153	Fully automated quantitative cephalometry using convolutional neural networks	2017	Arik SO et al.
3	106	Automated identification of cephalometric landmarks: Part I– Comparisons between the latest deep-learning methods YOLOV3 and SSD	2019	Park J et al.
4	104	Automated identification of cephalometric landmarks: Part II– Might it be better than human?	2020	Hwang H et al.
5	101	Artificial intelligence in orthodontics: Evaluation of a fully automated cephalometric analysis using a customized convolutional neural network	2020	Kunz F et al.
6	100	Tooth segmentation and labeling using deep convolutional neural network	2019	Xu X et al.
7	90	Artificial neural network modeling for deciding if extractions are necessary prior to orthodontic treatment	2010	Xie X et al.
8	86	Automated skeletal classification with lateral cephalometry based on artificial intelligence	2020	Yu HJ et al.
9	80	A fully automatic AI system for tooth and alveolar bone segmentation from cone-beam CT images	2022	Cui Z et al.
10	78	Artificial intelligence-driven novel tool for tooth detection and segmentation on panoramic radiographs	2021	Leite AD et al.

### Authors and collaboration analysis

Table 4 lists the top 8 authors for the number of published articles on the use of AI in orthodontics. In particular, the top 4 authors who published at least 10 articles were Kim Namkug, Kim Yoon-Ji, and Jacobs Reinhilde (the first for the number of citations equal to 329).

**Table 4:** Top 8 authors for the number of published articles on the use of AI in orthodontics

No	Count	Centrality	Year	Author	Citations
1	12	0.00	2019	Kim, Namkug	102
2	11	0.00	2020	Kim, Yoon-Ji	143
3	10	0.00	2021	Jacobs, Reinhilde	329
4	9	0.00	2020	Orhan, Kaan	180
5	8	0.00	2021	Van gerven, Adriaan	326
6	7	0.00	2021	Willems, Holger	313
7	7	0.00	2020	Xu, Tianmin Baek	57
8	7	0.00	2022	Turkkahraman, Hakan	32

### Country analysis

**Fig. 3:**
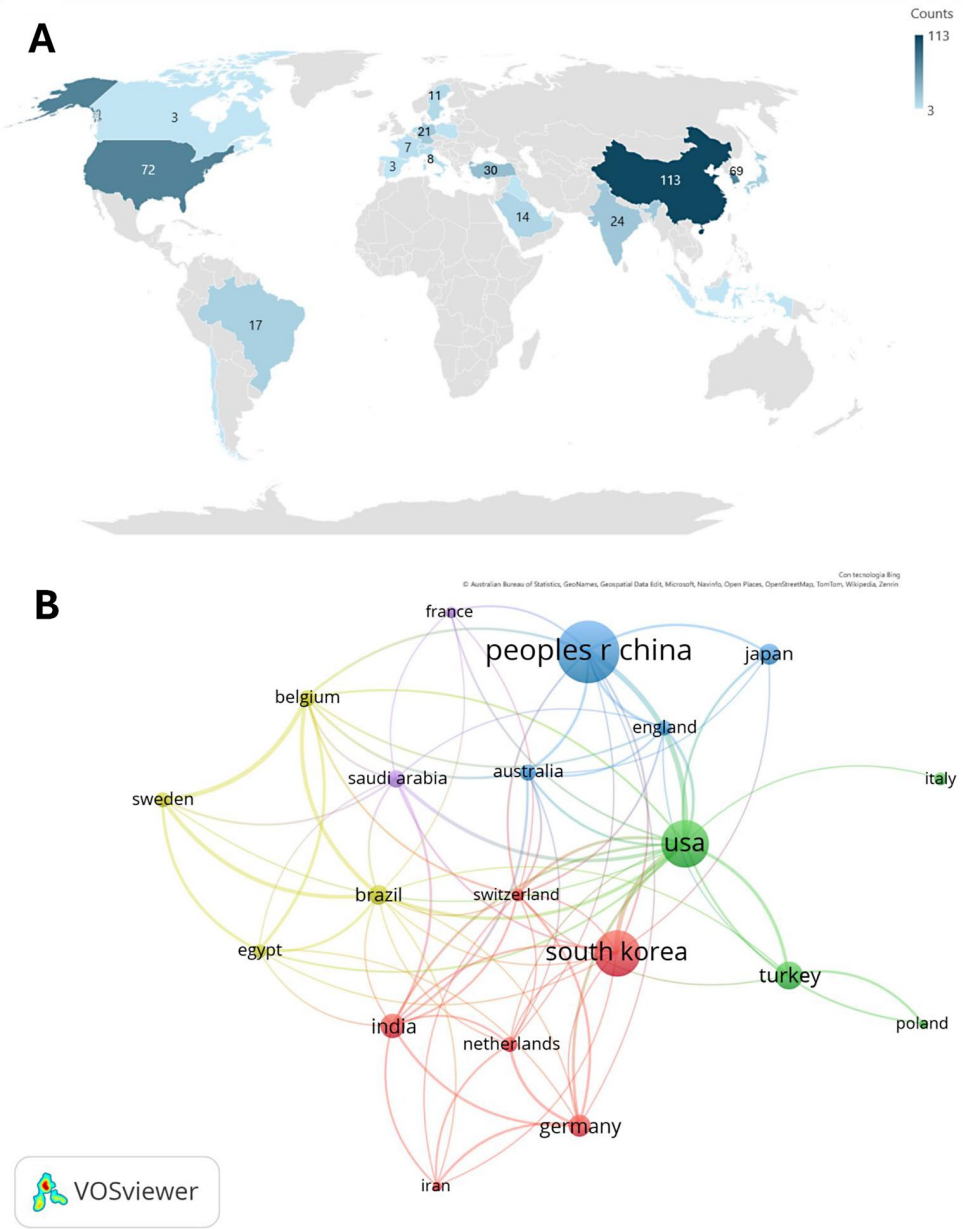
(**A**) Graphic representation of the top nations for the number of published articles on the use of AI in orthodontics; (**B**) Countries collaboration network

Regarding the countries involved in the scientific research for the use of AI in orthodontics, Table 5; Fig. 3B and C present the list of the top 10 countries classification based on the number of published articles. In particular, China is the first country in the world with a count of 113 papers and a high centrality (0.19), followed by the USA and South Korea with 72 and 69 articles, and 0.44 and 0.11 centralities respectively. Moreover, Fig. 3A shows a collaboration network that confirms the strict relationships between these 3 countries.

**Table 5:** Top 10 nations for the number of published articles on the use of AI in orthodontics. The “Year” column refers to the date of the first published study

No	Count	Centrality	Year	Countries
1	113	0.19	2010	China
2	72	0.44	2017	USA
3	69	0.11	2019	South Korea
4	30	0.00	2009	Turkey
5	24	0.13	2019	India
6	21	0.08	1998	Germany
7	18	0.00	2012	Japan
8	17	0.08	2005	Brazil
9	14	0.11	2019	Saudi Arabia
10	13	0.05	2021	Belgium

### Institution analysis

**Fig. 4:**
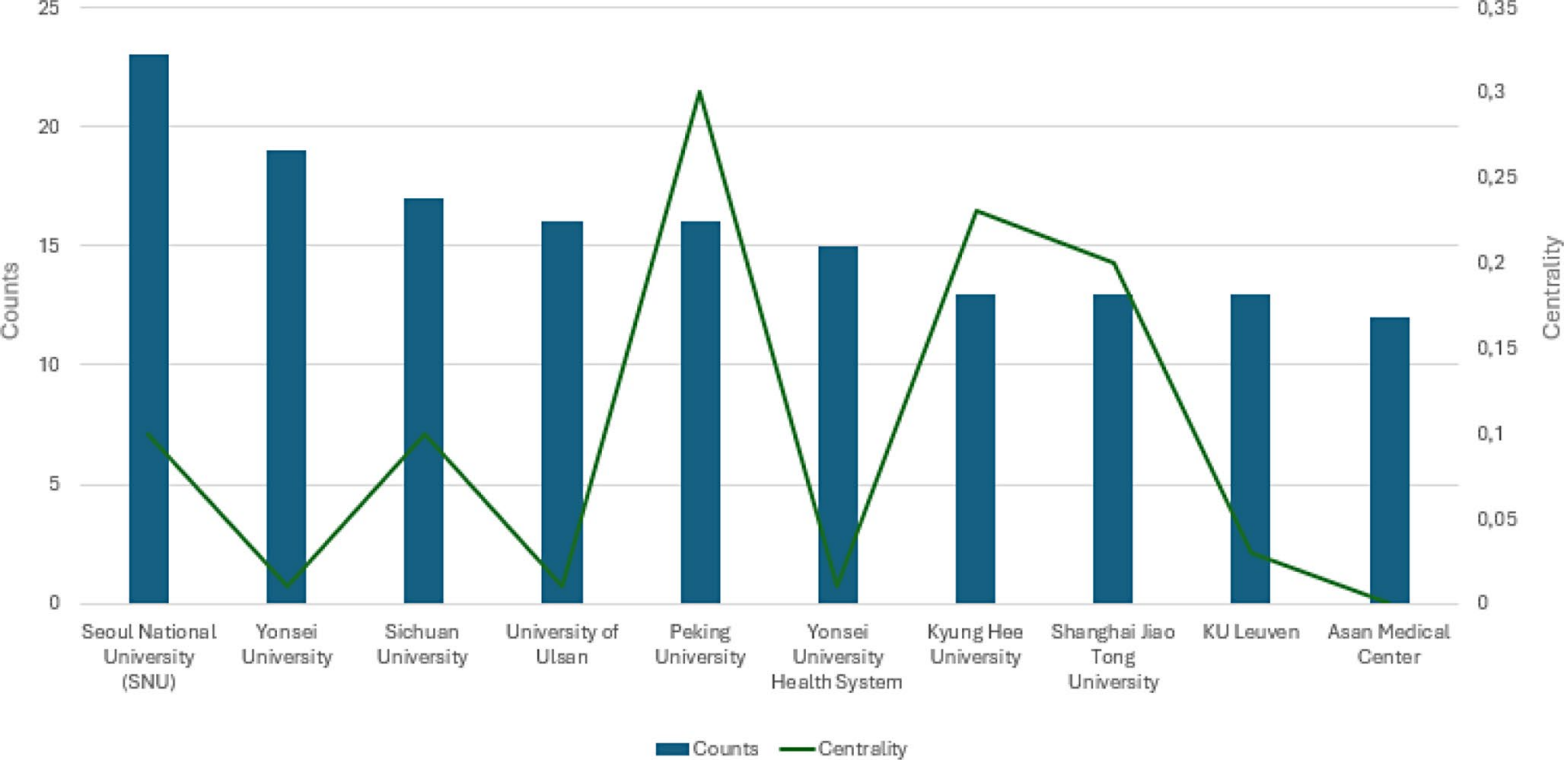
Publication trend per Institutions

Figure 4 shows the publication trend related to the involvement of Institutions. In particular, the Seoul National University stands out for the greatest number of published papers on the topic with 23 articles, followed by the Yonsei University, Sichuan University, the University of Ulsan, and Peking University with 19, 17, 16, and 16 published papers respectively. Moreover, Peking University and Hyung Hee University showed the highest centrality scores (0.30 and 0.23 respectively), indicating a high degree of collaboration with other institutions in scientific research related to the use of AI in orthodontics.

### Keywords analysis

#### Keywords co-occurrence

**Fig. 5:**
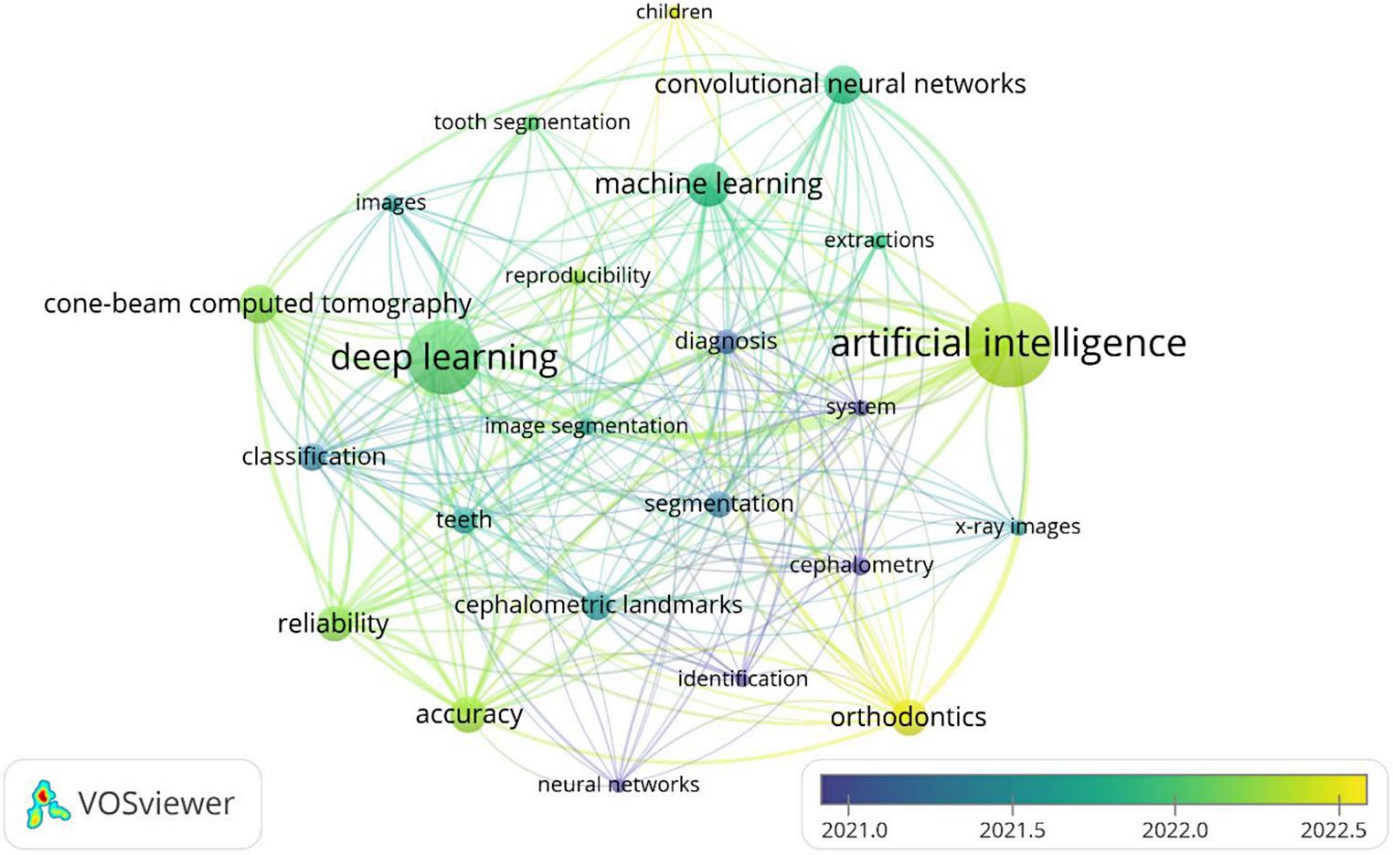
The co-occurrences of keywords with at least 15 apparitions

A total of 1203 keywords were identified. Figure 5A presents the most cited keywords with at least 15 occurrences and their relationships. In particular, the top-occurring keyword was “artificial intelligence” (137 occurrences, 0.50 centrality score), followed by (in order): “deep learning”, “machine learning”, “reliability”, “accuracy”, and “convolutional neural networks”. Most of these keywords were mainly cited starting from 2017 to 2019, and they are in concordance with the object of the present bibliometric analysis.

#### Keywords with citation burst

**Fig. 6:**
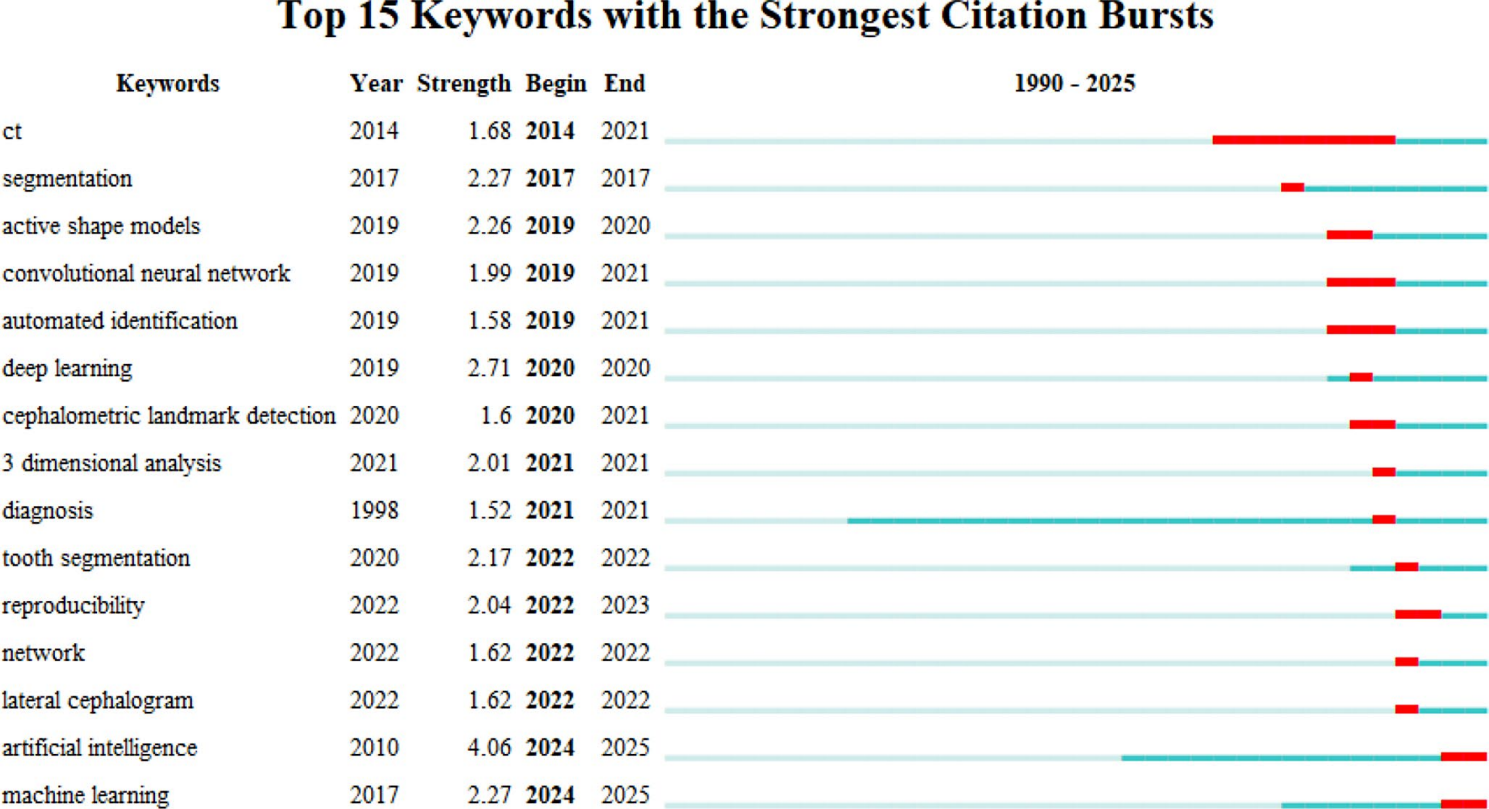
The top 15 keywords with the strongest bursts of citations; years with more frequent occurrences are indicated by the red line, and years with fewer appearances are indicated by the green line

Citation burst keywords illustrate the research hotspots in a certain field of study at various times by showing which keywords have a high citation rate during those periods [24]. Figure 6 shows the top 15 keywords with the strongest citation bursts. The top classified keywords in terms of strength were the following (in order): artificial intelligence (4.06), deep learning (2.71), machine learning (2.27), and segmentation (2.27). Artificial intelligence and machine learning have been characterized by frequent occurrences in recent years.

#### Keyword clustering

**Fig. 7:**
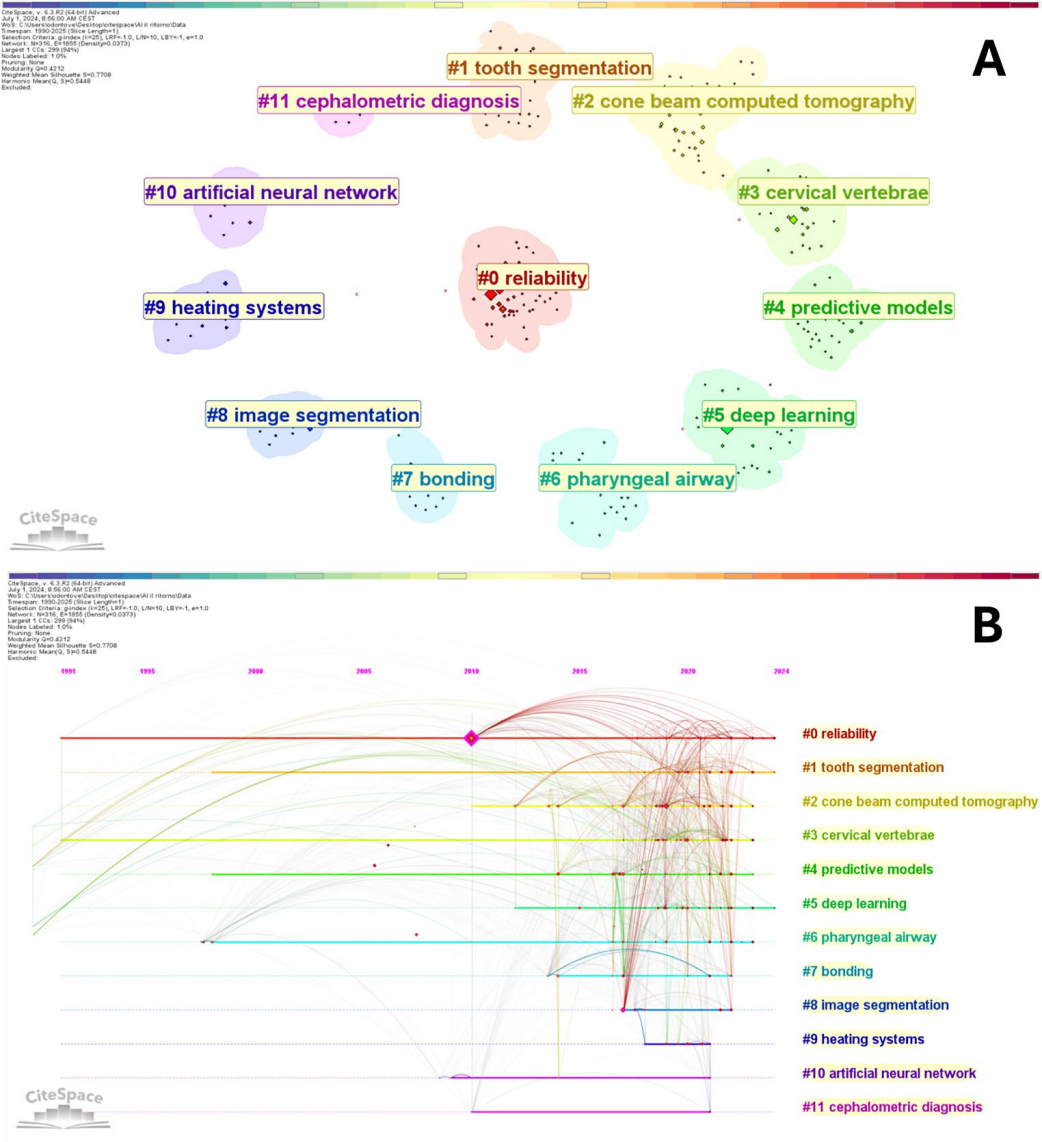
(**A**) Clustering arrangement of keywords; (**B**) clustering arrangement of keywords and their occurrence over the years

Ten different clusters were obtained after the extraction of cluster labels from terms associated with the titles of articles. Figure 7A and B shows the outcomes of the clustering with a modularity Q = 0.4212 and a weighted mean silhouette S = 0.7708, indicating a well-organized network and a very reliable clustering arrangement. Among the cluster labels, there are: “reliability”, “tooth segmentation”, “cone-beam computed tomography”, “cervical vertebrae”, “predictive models”, “deep learning”, “pharyngeal airway”, “image segmentation”, “artificial neural network” and “cephalometric diagnosis”. These clusters are aligned with the main topic of the present study.

## Discussion

The present bibliometric analysis aimed to provide a detailed overview of the current research trends and influential works tracing the line for future research directions. Moreover, this study identified the most impactful authors, articles, institutions and countries who contributed to the development of AI algorithms applied to orthodontics. Compared to the previous bibliometric analysis [13] on AI in orthodontics, we preferred not to emphasize CiteScore as a predictor of statistical quality, since it is a simple metric and not a quality index [25].

The results of the present analysis showed a growing and progressive interest in AI in orthodontics. In fact, the peak of publications was reached at the end of the last year (2023), indicating that this field is still in the full research and development phase, leaving new perspectives open to explore for clinical applications. Moreover, as previously mentioned, almost 83% of the available was published in the last 3 and a half years alone. This data is significant since it suggests that there is a growing interest in the scientific community in the research of AI to improve orthodontic diagnostic and therapeutic efficiency.

The journals that had the greatest impact on scientific publications in terms of citations obtained were specialist orthodontic and computer science journals; among these, the *American Journal of Orthodontics and Dentofacial Orthopedics*, *The Angle Orthodontist* and the *Lecturer Notes in Computer Science* stand out. A significant contribution in the field of AI in orthodontics derived also from journals not specialized in dentistry and orthodontics, since high centralities in different WoS categories related to electronic and informatic engineering, computer science, and medical imaging were observed. These results should not be surprising because AI applications on humans are characterized by a multidisciplinary interest that crosses the medical and computer science scientific community. In fact, recent attempts to promote standardised and comprehensive reporting of AI tool development and evaluation in healthcare have concentrated on offering guidelines for the reporting of AI studies for various medical domains or clinical tasks (e.g. TRIPOD + AI [26], CLAIM [27], CONSORT-AI [28], DECIDE-AI [29], PROBAST-AI [30], and CLEAR [31]). In other initiatives, the FUTURE-AI consortium [32], comprising 117 international and inter-disciplinary data science and medical experts from 50 countries, delivered the first structured and holistic guideline for trustworthy and ethical AI in healthcare, covering the entire lifecycle of AI.

According to the findings of the present study, it was evidenced that out of a total of 381 articles analyzed, about two thirds were led by China, USA, and South Korea, indicating that these three nations have had the greatest impact on the progress currently available in the use of AI in orthodontics. Similarly, the universities with the greatest number of published papers were the Seoul National University (South Korea), followed by the Yonsei University (South Korea), Sichuan University (China), the University of Ulsan (South Korea), and Peking University (China). Previous studies have proposed that disparities in research infrastructures, financing resources, languages, institutional support, and research interests could be the cause of these discrepancies in patterns among different countries [13, 33]. Government support is crucial for scientific progress and the growth of publications’ citation [34]. In this regard, China and the USA were the countries with the largest percentage of financed publications, with China reaching a peak of 87% [35]. Moreover, an additional explanation could be the large amount of population and search institutions of the USA and China.

The keywords analysis and clustering evidenced that automatic cephalometric analysis has been the main area of ​​research for AI in orthodontics with 78 counts of keywords related to this application over the last two decades. Over the two decades, many approaches involving knowledge-based, atlas-based, and learning-based algorithms have been used to automatically identify landmarks [36, 37]. In particular, the development of deep learning (DL), which uses neural networks to evaluate and interpret data, has accelerated the development of automatic cephalometric landmark detection [38]. In fact, the latest systematic reviews on this topic have shown an overall improvement in landmark identification when compared to the oldest [39–41]. However, despite this considerable number of studies on both 2D lateral cephalograms and 3D cone beam computed tomography (CBCT) images to shorten the time needed to obtain analysis, enhance the precision of landmark identification, and minimize errors related to the subjectivity of clinicians, it is still doubtful if AI could currently outperform manual landmarking in accuracy [42]. A systematic review by Schwendicke et al. [43] showed that the percentage of landmarks found within a 2 mm threshold in 3D imaging was greater (87%) than in 2D imaging (79.2%). Moreover, there was an imbalance in the automatic detection of the different landmarks. However, a recent umbrella review [44] pointed out that most of the studies available in the literature considered a wrong 2 mm cut-off in the difference between automatic and manual landmark identification, which is not clinically acceptable, and led to AI accuracy overestimation. As a result, the accuracy results for most AI models should be reinterpreted in the clinical context, since a variation of 1–2 mm in the location of various cephalometric points may significantly affect angular values as well as linear measurements, which in turn affects orthodontic diagnosis and treatment. Future studies will be necessary to refine new algorithms capable of reaching clinically acceptable landmarking discrepancies.

Automatic segmentation, another critical area in orthodontics, involves the delineation of anatomical structures from medical images [45]. Manual segmentation, considered the gold standard, is time-consuming and prone to inter-operator variability since the user segments the imaging data slice by slice [46]. AI-driven fully automatic segmentation tools have the advantage of enhancing the precision of identifying and isolating structures such as teeth, bones, and soft tissues in few seconds [47]. Despite these advantages, AI may be limited by the variability in image quality and the presence of artifacts, which can affect the accuracy of segmentation, especially for teeth. For example, many teeth in each arch can significantly complicate the segmentation process, especially when crowding is present. In addition, the tooth is a component made up of several tissues (cement, dentin, pulp, and enamel) that are close to various anatomical structures. Not all CBCT scanners can detect the thickness of the periodontal ligament, and the density of the alveolar bone is comparable to that of the tooth structure. Lastly, natural occlusion—difficulties in the separation of lower and upper teeth due to comparable densities—is the typical condition in which CBCT is obtained [48–51]. Convolutional Neural Networks (CNNs) were shown to be the most reliable method for automatic segmentation from CBCT [52]. However, to assess the dependability of the various DL architectures objectively, new research using standard methodologies and evaluation criteria including random sampling and blinding for data analysis is suggested [52].

Expert Systems (ES) decision-making ANNs-based have been developed to determine the necessity and strategy for tooth extractions during orthodontic treatment. AI systems can analyze patient data, including dental records and radiographs, to recommend extraction plans tailored to individual needs [53, 54]. This ES can help patients comprehend their treatment plans clearly in addition to helping students and less experienced orthodontists learn. To ascertain whether extractions are required before receiving orthodontic treatment, ES has been used [53, 54]. In this regard, the ES showed success rates ranging from 80 to 93% in accurately diagnosing extraction vs. non-extraction [54]. However, limitations arise from the reliance on comprehensive and high-quality data for training AI models. Incomplete or biased datasets can result in suboptimal extraction recommendations. A recent meta-analysis [55] showed that orthodontic tooth extraction decision-making using AI presented an overall good accuracy (0.87) with similar results with different algorithms, but the reliability of these results was lowered by high heterogeneity and very low certainty of evidence. Therefore, the orthodontists must ultimately make the final decision based on the clinical experience and the patient’s condition. Ongoing research on this topic, using sufficient sample sizes, models, validation, training, and testing sets supported by appropriate diagnostic references, was recommended to reinforce the accuracy of outcomes and clinical applicability [55].

AI models can analyze pre-treatment data to forecast post-treatment changes in soft tissues, aiding orthodontists in planning and managing patient expectations. In this regard, predictive models were created using ANN to forecast Class II patients’ changes in lip curvature following orthodontic treatment, with or without extractions [56]. A recent scoping review [57] including four non-randomized clinical trials found a low level of evidence indicating an estimated high overall accuracy of AI-generated prediction of facial changes after orthodontic treatment, whereas the lower lip and chin were the least predictable regions. Given the limited availability of studies, including non-randomized clinical trials with moderate-high risk of bias, more well-designed clinical trials with sufficient sample size have been recommended. Ensuring that AI predictions will be accurate and reliable across diverse patient populations will require extensive and diverse training datasets.

The results of the present bibliometric analysis showed that skeletal age assessment was another important field of investigation related to AI in orthodontics. In this regard, AI was evaluated to determine the optimal timing for interventions, in order to maximize treatment efficacy [58, 59]. For example, DL and CNNs were compared with human examiners for the classification of cervical vertebral maturation stages reaching an average accuracy of 71% [60]. However, it is still to be determined if these models may predict the variability in the developmental patterns among individuals. In fact, despite the high number of studies available in the literature, the diagnostic accuracy of AI models for cervical vertebral maturation assessment varied widely, ranging from 57 to 95%, due to the type of AI model, training data, and study methods [61]. AI accuracy was also influenced by the geographic concentration and variation in radiograph readers’ experience. Nonetheless, the lack of high-quality studies and the variation in AI performance point to the necessity for more investigation. In this regard, future studies with big datasets could increase the generalizability of AI algorithms’ performance.

Therefore, despite the significant advancements, AI in orthodontics is still in its nascent stages. The results of this study highlighted that the most explored field involved some areas of orthodontic diagnosis (i.e. automatic cephalometric analysis and skeletal age determination) [44, 52, 61, 62]. Instead, AI-driven treatment planning, the prediction of orthodontic treatment aesthetic outcomes, the need for orthodontic teeth extraction, growth prediction, mandibular and maxillary dental arch forms and the need for space to manage dental crowding are still under-explored areas that require studies with innovative methodology [55, 57]. Although the rapid evolution of AI in orthodontics was evidenced by a scoping review 3 years ago [3], the AI limitations in orthodontics still persist, despite the increased number of published studies on the topic and the improvement of technology during the last 3 years. Future research should focus on developing more robust and generalizable AI models by leveraging larger and more diverse datasets. Collaborative efforts across institutions can help in creating comprehensive databases that enhance the training and validation of AI systems. Furthermore, establishing clear guidelines for the development, validation, and deployment of AI tools in orthodontics will be crucial for ensuring their safe and effective use in the clinical setting.

When interpreting the results of the present study, some limitations should be addressed. The search strategy, although open and broad, may not have included the entirety of the literature available on the topic. However, the inclusion of the keyword “Orthodontic*” in the search strategy should have allowed the inclusion of the majority of relevant articles. Likely, most of the studies regarding the use of artificial intelligence in orthodontics should contain at least once the word “Orthodontic*” (and its derivatives) or “tooth/teeth”, “mandible” and “maxilla”. This is confirmed by the considerable number of articles included in this study and by the keyword analysis that highlighted the presence of the most important application areas in orthodontics. Patient education, which is a promising topic of AI application in orthodontics, was not considered in the aim of this study as it addressed the clinical practice. Moreover, for the sake of truth, the present bibliometric analysis was carried out on the Web of Science Core Collection only, which could represent a limitation. However, this was done according to previously published studies [9, 13, 63–65] and based on “CiteSpace” software compatibility characteristics [66]. Furthermore, the search was restricted to studies in the English language from 1985 onwards. However, this choice did not lead to the loss of significant data, since the oldest record dated back to 1991, and only 2 records were in German and 1 in Hungarian, whereas the remaining ones were published in English.

Finally, as every bibliometric analysis, this study did not assess the quality and reliability of included articles, because bibliometric analysis is a quantitative-approach study encompassing massive data useful for deciphering and mapping the cumulative scientific knowledge [5].

## Conclusions

Out of 381 selected articles, almost 83% were published in the last three and a half years, showing a rapid publication growth that is expected to be an ulterior increase. However, despite the growing interest, few original studies were conducted on AI-driven orthodontics. Although the majority of studies were published in highly impacted orthodontic journals, informatic engineering, computer science and medical imaging journals also provided a substantial contribution. Approximately a considerable amount of the available literature derived from authors and institutions from the USA, China and South Korea. Different areas of AI use in orthodontics were presented, showing that most of the research focused on AI-driven cephalometric landmarking and automatic segmentation.

The present bibliometric analysis offered a thorough overview of the current trend in orthodontics using AI, highlighted prominent research areas focused on increasing the speed and efficiency of orthodontic care and offered insight into potential directions for future research. Collaborative research efforts will be necessary to strengthen the maturity and robustness of AI models, which will be necessary to make AI-based clinical research sufficiently reliable for routine orthodontic practice.

## Data Availability

No datasets were generated or analysed during the current study.
